# Foliar Application of an Inositol-Based Plant Biostimulant Boosts Zinc Accumulation in Wheat Grains: A μ-X-Ray Fluorescence Case Study

**DOI:** 10.3389/fpls.2022.837695

**Published:** 2022-04-06

**Authors:** Douglas C. Amaral, Patrick H. Brown

**Affiliations:** ^1^Division of Agriculture and Natural Resources, University of California, Davis, Davis, CA, United States; ^2^Department of Plant Sciences, University of California, Davis, Davis, CA, United States

**Keywords:** biofortification, foliar fertilizer, biostimulant, wheat, zinc, μ-XRF

## Abstract

There has been much interest in the incorporation of organic molecules or biostimulants into foliar fertilizers with the rationalization that these compounds will enhance the uptake, or subsequent mobility of the applied nutrient. The objective of this research was to investigate the effects of an inositol-based plant stimulant on the mobility and accumulation of foliar-applied zinc (Zn) in wheat plants (*Triticum aestivum* L.). High-resolution elemental imaging with micro-X-ray fluorescence (μ-XRF) was utilized to examine Zn distribution within the vascular bundle of the leaf and whole grains. The inclusion of *myo*-inositol with Zinc sulfate, significantly increased Zn concentration in shoots in contrast to untreated controls and Zn sulfate applied alone. Foliar Zn treated plants increased Zn in grains by 5–25% with *myo*-inositol plus Zn treated plants significantly increasing grain Zn concentration compared to both Zn treated and non-treated controls. XRF imaging revealed Zn enrichment in the bran layer and germ, with a very low Zn concentration present in the endosperm. Plants treated with Zn plus *myo*-inositol showed an enhanced and uniform distribution of Zn throughout the bran layer and germ with an increased concentration in the endosperm. While our data suggest that foliar application of *myo*-inositol in combination with Zn may be a promising strategy to increase the absorption and mobility of Zn in the plant tissue and subsequently to enhance Zn accumulation in grains, further research is needed to clarify the mechanisms by which *myo*-inositol affects plant metabolism and nutrient mobility.

## Introduction

Wheat (*Triticum aestivum* L.) is a cereal grass widely cultivated for their edible grains. Among widely cultivated food crops, wheat plays a particularly important role in daily energy intake, especially in the developing world. Modern wheat cultivars are however a poor source of micronutrients (e.g., Zn and Fe) and when used as a dominant part of daily calorie intake, a wheat based diet often fails to meet human Zn and Fe requirements (Cakmak et al., 2010). To meet human dietary needs in wheat dominant diets, it is estimated that grain Zn concentrations should be increased from the current average of 20–35 mg kg^–1^ to greater than 50 mg kg^–1^ (Cakmak et al., 2010). Foliar fertilization can be used to effectively enrich Zn grain though the efficiency of transport of foliar applied Zn to grain is generally low (Erenoglu et al., 2011).

Foliar application of nutrients in crop plants is an important agricultural practice world-wide (Fernández et al., 2013). Foliar fertilization is essential where soil conditions such as high pH, limit the availability of soil applied fertilizers and where there is a need to ensure nutrient adequacy at critical stages of plant growth (Tian et al., 2015). Many factors may influence the efficacy of foliar fertilizers such as the physiological status and phenological stage of the plant, the mobility of the nutrient within the plant, or the presence of abiotic stresses (Fernández and Brown, 2013; Fernández et al., 2013). For example, Zn fertilizers, when applied *via* foliar fertilization, must readily penetrate the leaf and remain soluble to promote the translocation from leaves to phloem-fed tissues (White and Broadley, 2011; Saa et al., 2018). However, it has been reported that foliar application of common sources of Zn such as Zinc sulfate (ZnSO_4_) and Zn-EDTA may not improve the mobility of Zn in plant tissues (Doolette et al., 2018). Thus, enhancing the efficiency of the foliar applied nutrients by adopting new strategies is of great importance not only from an economic, agronomic and environmental point of view, but also as a means to correct human nutrient deficiencies.

The use of plant biostimulants, such as inositol compounds, is emerging as a central feature in plant biochemistry and physiology (Yakhin et al., 2017). Inositol which is well known for acting as a stress-ameliorator in plants (Hu et al., 2018; Mukherjee et al., 2019) may also control multiple aspects of plant signaling and physiology (Jia et al., 2019). Previous reports suggest that exogenous application of inositol could not only alleviate stress but also alter gene expression involved in cell wall biosynthesis, regulation of phytohormones, redox reactions, and chromosome modifications (Chatterjee and Majumder, 2010; Donahue et al., 2010; Ye et al., 2016). In addition, some inositol isoforms (e.g., *myo*-inositol) may act as a strong chelator of metal cations possibly facilitating absorption and transport of nutrients. Given the important role of inositol in cellular functioning, especially its protective functions under biotic and abiotic stresses, surprisingly little is known about exogenous application of inositol in plants such as responses to nutrient availability and effects on mobility.

The development of new techniques that will help improve the efficacy of foliar applied nutrients by enhancing the absorption, translocation, and its utilization by plants with the ultimate goal of improving the quality and yields of crops is mandatory. Due to its capacity to potentially facilitate ion transmembrane transport, we hypothesize that *myo*-inositol applied in combination with Zn may increase its absorption, transport, and accumulation in plant tissues. Here, our goal was to provide comprehensive information on the mobility and distribution of foliar applied zinc, with or without *myo*-inositol, in wheat leaves and its accumulation and localization in grains using X-ray fluorescence (XRF) synchrotron-based techniques which represents a powerful tool for characterizing *in vivo* nutrient mobility and distribution in plants.

## Materials and Methods

### Plant Culture

Sterilized wheat seeds (*Triticum aestivum* L., cv. Patwin 515) were soaked in deionized water for 24 h and then germinated in containers filled with a soil mixture containing (% of volume) 40% peat, 35% silica clay, 20% perlite, and 5% gravel. 10-day-old seedlings of uniform height were transplanted into each 5L pot, one plant per pot, and the pots were transferred to a controlled environment greenhouse with day/night temperature of 25/20°C and day/night humidity of 70/85%. Lighting was provided by LED grow lights with 16-h day length. Plants were watered as needed with a nutrient solution [1.2 mM KNO_3_, 0.8 mM Ca(NO_3_)_2_, 0.8 mM NH_4_NO_3_, 0.3 mM KH_2_PO_4_, and 0.2 mM MgSO_4_, 12 μM Fe-EDTA, 0.25 μM Na_2_B_8_O_13_⋅4H_2_O, 1.5 μM MnSO_4_, 0.25 μM ZnSO_4_, 0.5 μM CuSO_4_, and 0.04 μM Na_2_MoO_4_]. Each treatment and analyses were conducted in four biological replicates.

### Foliar Treatments

During stem elongation (flag leaf ligule and collar visible) plants were treated with the following treatments: water sprayed control (no added Zn, “*-Zn-CK*”), zinc sulfate control (added Zn, “+*Zn-CK*”), *myo*-inositol (no added Zn, “*-Zn*+*Ino*”), and *myo*-inositol plus zinc sulfate (added Zn, “+*Zn* +*Ino*”). ZnSO^–4^ was applied at the final concentration of 250 mg L^–1^, and *myo*-inositol was applied at the concentration of 0.25% v/v. The selected concentration was based on previous experiments (data not shown). *Myo*-inositol was selected due to its capacity to potentially facilitate ion transmembrane transport. The treatments were applied to the leaves of wheat 8 h before darkness (10 a.m.). The soil was covered to prevent inadvertent spray drift. Foliar application was repeated 15 days after initial application. To avoid the possibility that the effect of the foliar spray was a consequence of alleviation of secondary nutrient deficiency, all plants were grown with continuous and abundant soil nutrient.

### Elemental Analysis

At plant maturity, shoot and grains were collected and prepared for elemental analysis. The wheat grains were removed from panicles and dehusked, and several dehusked grains were set aside for X-ray fluorescence (XRF) imaging. Leaves and grains were oven-dried at 65°C for 72 h and ground using a stainless-steel mill (0.2-mm screen). Ground, dry plant samples (0.1 g) were digested with concentrated trace-metal grade (TMG) HNO_3_. Total concentrations of elements (i.e., Zn, Fe, Cu, and Mn) in the filtrates were analyzed using inductively coupled plasma mass spectroscopy (ICP-MS; Agilent 7500a, United States). Standard Reference Material (SRM) Tomato Leaves (NIST 1573a) were used to verify the results and gave excellent recoveries for all the elements of interest (≥94%).

### Elemental Mapping

#### μ-X-Ray Fluorescence Sample Preparation

Mid-sections of leaves were obtained from plants treated with different foliar treatments. Leaf cross-sections (30 μm) were sectioned with a cryotome (LEICA, CM1850) at a temperature of −20°C. Single sections of each treatment were selected under light microscopy for their ultrastructural integrity and then freeze-dried under −20°C for 3 days prior to XRF analysis. The dehusked grains were set in epoxy (EPO-TEK 301-2FL) resin, cured, and cut into thin sections.

#### μ-X-Ray Fluorescence Analyses

X-ray fluorescence imaging of the grain and leaf thin sections was conducted at the Stanford Synchrotron Radiation Laboratory (SSRL) at beam lines 10–2 and 2–3. Experiments at Beam Line 10–2 were recorded at 10500 eV, using a 20 μm (H) × 20 μm (V) beam spot size, a 20 μm × 20 μm pixel size, and 200 ms dwell time per pixel. The incident X-ray beam of 2 μm at beam line 2–3 was focused using a pair of Kirkpatrick–Baez mirrors, and the incident beam was monochromatized using a Si(111) double-crystal monochromator. Micro-XRF maps were obtained by rastering the beam at 5 μm steps, with a count time of 100 ms per step. Fluorescence intensities of P, S, Cl, K, Ca, Mn, Fe, Ni, Cu, and Zn were monitored. Fluorescence signal intensities for Zn and any other elements were calculated with SMAK (Sam’s Microprobe Analysis Kit, Version 1.5).

### Experimental Design and Statistical Analysis

The experimental design was a randomized complete design with a full factorial structure. Each treatment and analyses were conducted in four biological replicates. Analysis of variance (ANOVA) was performed with SigmaPlot (Systat Software, Inc., Version 14.0). If significant differences (*P* < 0.05) were identified, a Tukey test was used to distinguish differences between the groups.

## Results

### Grain Yield and Plant Biomass

**FIGURE 1:**
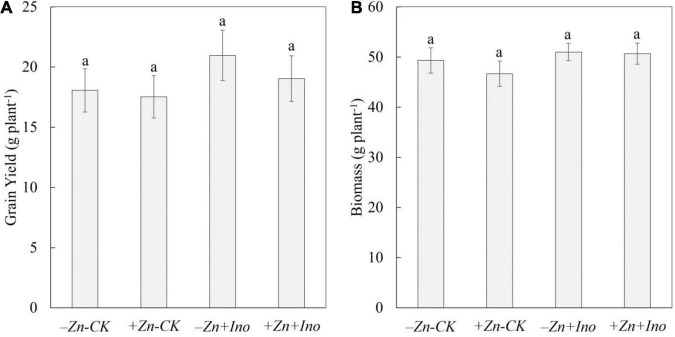
Grain yield **(A)** and plant biomass **(B)** of wheat treated with different foliar fertilizers. Data points and error bars represent means and SE of four replicates. Different letters denote significant differences (*p* < 0.05, *n* = 4).

Plant biomass and yield were determined at full plant maturity (Figure 1). Treatments did not significantly affect plant biomass (i.e., straw) or yield (i.e., whole grain mass) however the use of *myo*-inositol increased grain yield on average by up to 15% compared to -Zn-CK.

### Total Zn Concentration in Shoots and Grains

**FIGURE 2:**
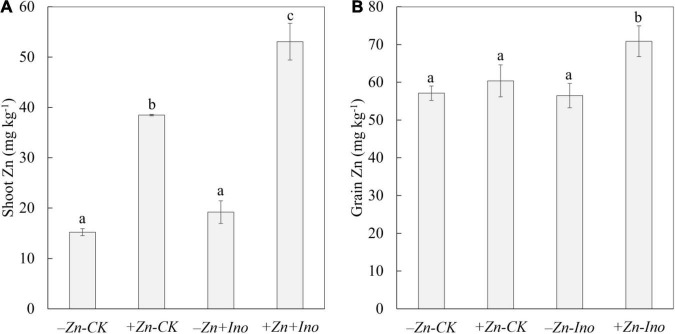
Zinc (Zn) concentrations in shoots **(A)** and grains **(B)** of wheat plants treated with different foliar fertilizers. Data points and error bars represent means and SE of four replicates. Different letters denote significant differences (*p* < 0.05, *n* = 4).

All treatments affected shoot zinc concentrations (Figure 2). Shoot Zn concentrations ranged from 15.2 to 53.1 mg kg^–1^ and were significantly affected by both Zn treatments, with +Zn+Ino treated plants having significantly more zinc than the non-treated control (-Zn-CK) by nearly fourfold and significantly increasing shoot Zn by 13 mg kg^–1^ approximately 40% in contrast to +Zn-Ino treatments. Total zinc concentrations in shoots were lowest for the control and *myo*-inositol treated plants, while shoot zinc in plants treated with zinc alone or applied with *myo*-inositol was significantly higher (*p* = 0.001).

Similar, though smaller trends were observed in the grains where Zn-treated plants both with and without *myo*-inositol had increased zinc concentration compared with non-treated control (Figure 2). In the absence of added zinc, *myo*-inositol had no effect on grain zinc levels, with the addition of both *myo*-inositol and Zn grain zinc levels increased by 20 ppm (*p* < 0.05). Zinc values in unpolished grains ranged from 55.5 to 75.6 mg kg^–1^ for -Zn-CK and +Zn+Ino treatments, respectively. On average, Zn treated plants increased zinc in grains by 5–25% with +Zn+Ino significantly increasing grain zinc concentration compared to both Zn-treated (*p* = 0.018) and non-treated controls (*p* = 0.003).

### Distribution Patterns of Zinc in Leaf and Grain of Wheat Plants

Micro-XRF mapping was performed to investigate the effects of different foliar applied Zn and *myo*-inositol treatments on the distribution and localization of zinc in leaves of wheat at full plant maturity. The cross sections of wheat leaves are composed of upper and lower epidermis, parenchyma, and vascular tissue (xylem and phloem). Spatial imaging of zinc was also performed on cross-sections of unpolished wheat grains collected during plant harvest. The cross sections of unpolished wheat grains are composed of bran layer, endosperm, and germ. The normalized XRF intensities of each map indicates the relative distribution for each individual element.

**FIGURE 3:**
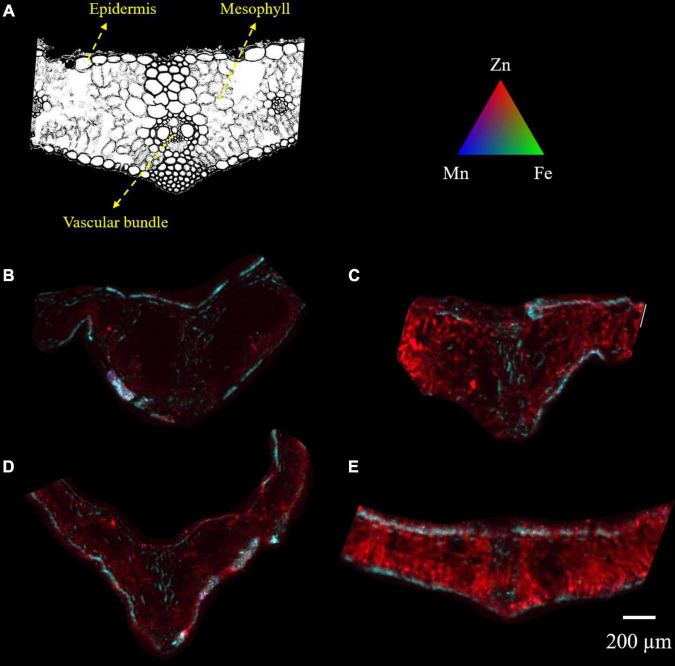
Microscope cross section **(A)** and μ-XRF elemental maps **(B–E)** for Zn (red), Fe (green), and Mn (blue) of wheat leaf treated with different foliar fertilizers: −Zn-CK **(B)**, +Zn-CK **(C)**, −Zn+Ino **(D)**, and +Zn+Ino **(E)**. Pixel brightness for μ-XRF map **(B–E)** is displayed in RGB, with the brightest spots corresponding to the highest elemental fluorescence. Scale bar: 200 μm. *n* = 4, representative images shown.

X-ray fluorescence images showed that wheat leaves exhibited differential zinc distribution on plant tissue with a higher degree of distribution to the mesophyll and vascular tissues (Figure 3). When adjusted to the same relative scale, the zinc distribution on the +Zn-CK and +Zn+Ino treated plants was much more extensive and higher in fluorescence intensity than on the −Zn-CK and −Zn+Ino treated plants with +Zn+Ino treated plants showing a higher intensity when compared to +Zn-CK treated plants. This finding corroborates with the ICP-MS data (Figure 2), suggesting that *myo*-inositol applied *via* foliar spray in combination with Zn may facilitate solute movement into plant cells.

**FIGURE 4:**
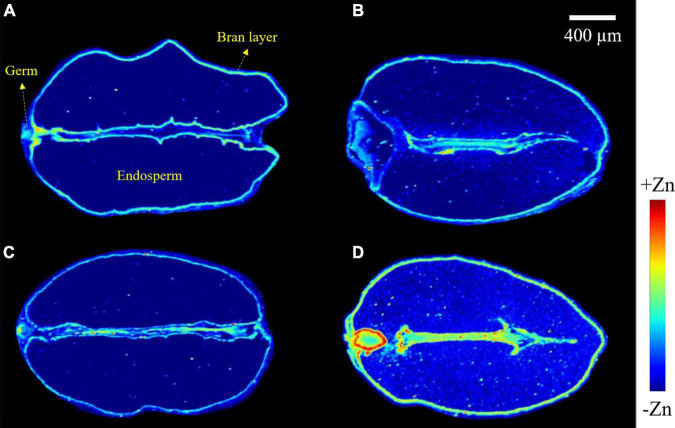
Micro X-ray fluorescence (μ-XRF) elemental maps for Zn of grains collected from wheat plants subject to different foliar fertilizers: −Zn-CK **(A)**, +Zn-CK **(B)**, −Zn+Ino **(C)**, and +Zn+Ino **(D)**. Pixel brightness for μ-XRF maps **(A–D)** is displayed in RGB, with the brightest spots corresponding to the highest elemental fluorescence. Scale bar: 400 μm. *n* = 4, representative images shown.

Images obtained from a longitudinal cross-section of wheat grains show the distribution of zinc (Figure 4). In −Zn-CK and −Zn+Ino treatments, zinc was distributed in highest concentration in the bran layer and germ, with a very small concentration present in the endosperm. For +Zn-CK and +Zn+Ino treated plants, zinc was also present in highest concentration in the bran layer and germ, but exhibited an increased concentration in the endosperm, with +Zn+Ino showing an enhanced and uniform distribution of zinc throughout the grain. Furthermore, intensity analysis across a single scan line through the grain confirmed that the peak of Zn intensities in the bran layer, endosperm, and germ was noticeably increased in the +Zn+Ino treatment.

All treatments showed the same pattern of distribution for other plant nutrients (data not shown). Iron, manganese, and copper follow a similar allocation pattern as zinc. Calcium was distributed throughout the grain with highest levels in the germ and bran layer, and lowest in the endosperm. Potassium and phosphorus follow a similar distribution pattern as calcium, with potassium being more abundant in the endosperm.

### Plant Nutrients

**FIGURE 5:**
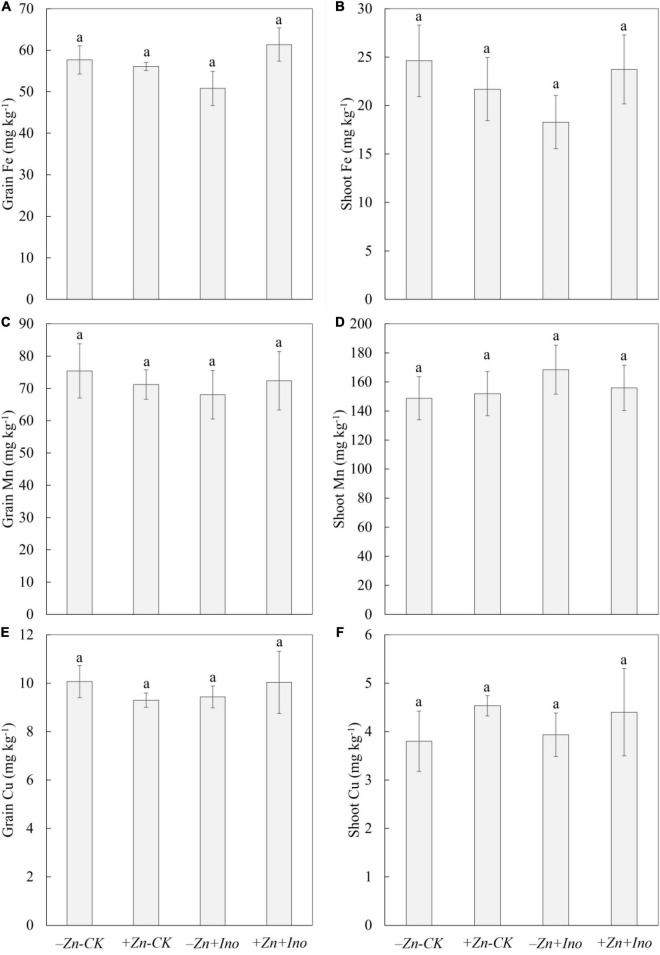
Concentrations of Fe **(A,B)**, Mn **(C,D)**, and Cu **(E,F)** in leaves and grains of wheat plants treated with different foliar fertilizers. Data points and error bars represent means and SE of four replicates (*p* < 0.05, *n* = 4).

Of the other plant nutrients analyzed (i.e., Fe, Mn, and Cu), none were significantly affected by the treatments compared to the −Zn-CK suggesting that foliar application of Zn and *myo*-inositol, alone or in combination, may increase total grain zinc in wheat without negatively affecting grain Fe, Mn, or Cu (Figure 5).

In the shoots, +Zn-CK treatment increased the concentration of Mn (155.9 mg kg^–1^) and Cu (4.5 mg kg^–1^), and slightly decreased Fe (21.7 mg kg^–1^) concentration compared to the −Zn-CK (Mn: 148.8 mg kg^–1^; Cu: 3.8 mg kg^–1^, and Fe: 24.6 mg kg^–1^). −Zn+Ino also enhanced Mn (151.9 mg kg^–1^) and Cu (3.9 mg kg^–1^), and reduced Fe concentration (18.3 mg kg^–1^). +Zn+Ino increased Mn concentration by 13% (168.5 mg kg^–1^) and Cu by 15% (4.4 mg kg^–1^) but did not affect Fe concentration (23.7 mg kg^–1^).

In grains, +Zn-CK, −Zn+Ino, and +Zn+Ino decreased Mn concentration by 5% (71.2 mg kg^–1^), 10% (68.0 mg kg^–1^), and 4% (72.4 mg kg^–1^), respectively, compared to the −Zn-CK (75.4 mg kg^–1^). Fe and Cu concentration in −Zn-CK, +Zn-CK, −Zn+Ino, and +Zn+Ino treatments were consistent showing concentrations of 57.7 and 10.1 mg kg^–1^, 56.1 and 9.3 mg kg^–1^, 50.8 and 9.4 mg kg^–1^, and 61.4 and 10.0 mg kg^–1^, respectively.

## Discussion

Zinc sulfate (ZnSO_4_) and Zn-EDTA are two common forms of foliar-applied Zn fertilizers (White and Broadley, 2009, 2011). However, low or limited mobility of zinc in leaves when applied in the form of SO_4_ (Tian et al., 2015) or chelated with EDTA (Doolette et al., 2018) has been reported. To improve the efficacy of foliar applied Zn, we focused on developing new strategies aiming to increase Zn transport from leaf surface to phloem-fed tissues and subsequently improving its transport and accumulation in grains.

Results from the present study showed clearly increased accumulation of Zn in both leaf and grains of wheat after the application of ZnSO_4_ in combination with *myo*-inositol, while ZnSO_4_ applied alone increased shoot Zn significantly but had only slightly increased Zn distribution and accumulation in grains (Figures 2–4). *Myo*-inositol applied alone slightly increased shoot Zn concentrations (Figure 2A) suggesting this product may also influence root Zn uptake or Zn transport of previously acquired Zn, this effect is being examined in separate experiments. The significant positive effect of +Zn+Ino on shoot (Figure 2A) and grain (Figure 2B) Zn concentrations and changes within organ Zn distribution suggests *myo*-inositol may alter both Zn penetration into the shoot tissue and the subsequent intercellular mobility of acquired Zn. While Zn has been reported as having a poor leaf penetration and low mobility in the phloem (White and Broadley, 2011), this study showed evidence that *myo*-inositol may improve Zn mobility in the plant tissue (Figure 3) resulting in greater phloem Zn transport, and higher grain Zn concentrations (Figure 4) than ZnSO_4_ alone.

The role of inositol in plant metabolism is uncertain. Inositol may function in various plant signaling pathways, such as energy homeostasis, phosphate sensing, and immune responses (Williams et al., 2015). However, its existence and function in plants is newly emerging and little is known about its use as a plant stimulant. Inositol has been proposed to be an important component for stomatal regulation in plants as it is implicated in abscisic acid-mediated signaling (Perera et al., 2008). Recently, (Hu et al., 2018) showed that exogenous application of *myo*-inositol not only improved the photosynthetic activity and plant growth in *Malus hupehensis* plants, but also up-regulated stomatal apertures allowing those plants to modulate transpirational water losses and the uptake of CO_2_. While the mechanisms of absorption of solutes through the stomata are not very well understood (Fernández and Brown, 2013), it can be highly significant as the number of stomata contributing to uptake proved to be highly variable in the leaf surface (Eichert et al., 2008). For example, in *Vicia faba*, exogenously administered phosphatidyl-inositol induced stomatal opening and swelling in guard cell protoplasts (Lee et al., 2007). Thus, by up-regulating stomatal apertures, *myo*-inositol application may result in an improved distribution of permeability on the leaf surface possibly enhancing the diffusion of solutes into the stomatal pores. Li et al. (2019) studying the pathways in which foliar-applied ZnSO_4_ moves through the sunflower leaf surface, considering the potential importance of the cuticle, stomata and trichomes, demonstrated that there is no indication that the stomata played a major role in the foliar absorption of ZnSO_4_. This corroborates our hypothesis where ZnSO_4_ applied alone increased Zn in plant tissue only by twofold, while *myo*-inositol applied in combination with ZnSO_4_ in the same concentration (250 mg L^–1^) enhanced plant Zn by nearly fourfold compared to the control, possibly by modulating stomatal opening and increasing Zn absorption by the leaf.

An oxidized form of inositol is also the most common and important sugar implicated in polysaccharide synthesis for cell walls (Loewus and Murthy, 2000; Cui et al., 2013). In addition to cell wall biosynthesis, inositol and its derivatives provide components for other vital pathways such as, but not limited to, biotic and abiotic stress response, plant growth and development, signaling transduction, and substrate transportation (Ye et al., 2016). Some recent studies have reported the benefits of non-foliar applied *myo*-inositol in plants under different conditions (Liu et al., 2006; Lee et al., 2007; Perera et al., 2008; Zhai et al., 2016; Belgaroui et al., 2018; Mukherjee et al., 2019). For example, in apple rootstock *Malus hupehensis*, root-applied *myo*-inositol alleviates salt-induced inhibition of growth not only acting in antioxidant defenses, but also mediating Na^+^ and K^+^ homeostasis and the osmotic balance (Hu et al., 2018). In contrast, very little is known regarding foliar application of *myo*-inositol and its possible benefits in plants. It has been reported that Zn is coordinated to P as Zn-Phytic acid (inositol-6-phosphate), which is composed of six Pi molecules linked to a *myo*-inositol, in roots and in leaves of *Arabidopsis lyrata* (Sarret et al., 2003; Bouain et al., 2014). According to Bouain et al. (2014), the occurrence of co-regulation of Pi–Zn nutrition in cells of intact plants may impact both P and Zn uptake and transport within a plant body. When exposed to a combination of ZnSO_4_ and *myo*-inositol, wheat plants showed an increased accumulation of Zn in both shoots and grains. We speculated that the increased Zn concentration in response to *myo*-inositol application might be a gene-mediated response strategy related to ion transmembrane transport, leading to absorption of Zn as it has recently been shown that exogenous *myo*-inositol could alter gene expression and signaling transduction in plant cells (Ye et al., 2016). Furthermore, several genes related to substance transport in response to *myo*-inositol *application* were identified suggesting that *myo*-inositol might act as a regulator mediating the transportation of carbohydrates and other substances. However, no further evidence on its implication in the regulation of Zn transport and/or signaling has been discovered. Therefore, further research should focus on the role of *myo*-inositol in co-regulating Zn transport and signaling crosstalk.

On the basis of existing knowledge of inositol function in plants we propose two main mechanisms to explain the increased plant Zn accumulation and transport due to *myo*-inositol application (1) *myo*-inositol may have changed plant stomatal regulation and increased Zn absorption by wheat leaves; and (2) *myo*-inositol may have changed cell permeability by gene-mediated response strategy related to ion transmembrane transport, facilitating Zn absorption by the leaf. To our understanding, this is the first study utilizing μ-XRF to show visual evidence for the *in situ* investigation of Zn in wheat leaves and grains after foliar application of *myo*-inositol. We found that *myo*-inositol significantly affected Zn transport and distribution in leaf and grains of wheat. Additionally, *myo*-inositol treated plants increased plant yield by 5–15% compared to the controls. The results suggested that *myo*-inositol could affect the physiological status of plant cells and might also influence the mobility of Zn in the plant tissue. The observations above introduce many important points considering the form in which foliar applied Zn penetrates the leaf and how Zn is redistributed in leaf tissue when applied in combination with *myo*-inositol. While further research is required to better understand the specific absorption or uptake pathway of different foliar Zn fertilizers and/or plant stimulants, the results show evidence that *myo*-inositol may improve the efficiency of foliar-applied ZnSO_4_.

## Conclusion

The diet of much of the world’s population is based on cereals such as wheat that contain insufficient amounts of numerous nutrients (e.g., Zn, Fe, Cu, and Mn) to meet daily needs. Our results showed that foliar application of *myo*-inositol in combination with Zn may be a promising strategy to increase the absorption and mobility of Zn in the plant tissue and subsequently to enhance Zn accumulation in grains without affecting other essential metals such as Fe, Mn and Cu or decreasing grain yield. Further research is needed to clarify the mechanisms by which *myo*-inositol affects plant metabolism and to identify stimulant properties that can considerably increase grain micronutrients under a wide range of field conditions to maximize plant response to foliar applications.

## Data Availability Statement

The original contributions presented in the study are included in the article/supplementary material, further inquiries can be directed to the corresponding author.

## Author Contributions

DA and PB designed the study. DA performed the experiments, analytical determinations, carried out the data analyses, and wrote the first draft of the manuscript. PB was responsible for the revision of the final manuscript. Both authors have given final approval for this version of the manuscript.

## Conflict of Interest

The authors declare that the research was conducted in the absence of any commercial or financial relationships that could be construed as a potential conflict of interest.

## Publisher’s Note

All claims expressed in this article are solely those of the authors and do not necessarily represent those of their affiliated organizations, or those of the publisher, the editors and the reviewers. Any product that may be evaluated in this article, or claim that may be made by its manufacturer, is not guaranteed or endorsed by the publisher.
